# How are poor sleepers with other clinical conditions affected by maladaptive personality traits? A neural network-based analysis

**DOI:** 10.3389/fpsyt.2024.1392525

**Published:** 2024-07-12

**Authors:** Habibolah Khazaie, Farzin Rezaei, Ali Zakiei, Behrooz Faridmarandi, Saeid Komasi

**Affiliations:** ^1^ Sleep Disorders Research Center, Kermanshah University of Medical Sciences, Kermanshah, Iran; ^2^ Department of Psychiatry, Roozbeh Hospital, Tehran University of Medical Sciences, Tehran, Iran; ^3^ Department of Neuroscience and Psychopathology Research, Mind GPS Institute, Kermanshah, Iran

**Keywords:** depression, machine learning, maladaptive trait, personality disorder, sleep quality, somatization

## Abstract

**Background:**

Psychopathology research mainly focused on the cross-sectional and longitudinal associations between personality and psychiatric disorders without considering the moment-to-moment dynamics of personality in response to environmental situations. The present study aimed to both cluster a young sample according to three mixed clinical conditions (poor sleep quality, depression, and somatization) and to predict the derived clusters by maladaptive personality traits and sex differences using a deep machine learning approach.

**Methods:**

A sample of 839 adults aged 18-40 years (64% female) from the west of Iran were clustered according to the mixed clinical conditions using the cluster analysis techniques. An Artificial Neural Network (ANN) modeling is used to predict the derived clusters by maladaptive personality traits and biological gender. A receiver operating characteristic (ROC) curve was used to identify independent variables with high sensitivity specific to the derived clusters.

**Results:**

The cluster analysis techniques suggested a fully stable and acceptable four-cluster solution for Depressed Poor Sleepers, Nonclinical Good Sleepers, Subclinical Poor Sleepers, and Clinical Poor Sleepers. The ANN model led to the identification of one hidden layer with two hidden units. The results of Area under the ROC Curve were relatively to completely acceptable, ranging from.726 to.855. Anhedonia, perceptual dysregulation, depressivity, anxiousness, and unusual beliefs are the most valuable traits with importance higher than 70%.

**Conclusion:**

The machine learning approach can be well used to predict mixed clinical conditions by maladaptive personality traits. Future research can test the complexity of normal personality traits connected to mixed clinical conditions.

## Introduction

Sleep is a complex physiological phenomenon that maintains homeostasis in the human organism and is regulated globally and locally by both cellular and molecular mechanisms (1). Sleep quality is characterized by an individual’s level of self-satisfaction with all aspects of the sleep experience (2). Because poor sleep quality contributes to disease and poor health outcomes including fatigue, slowed responses, irritability, and daytime dysfunction, it is currently a global health concern (2). However, poor sleep quality is usually not an independent clinical condition and is relatively mixed with other psychiatric conditions such as depression (3–5). For example, the results of a study showed that poor sleep quality is a significant risk factor for depression (4). Poor sleep quality can also be comorbid with other clinical conditions such as somatization (3, 6). Nevertheless, poor sleep quality is not only a risk factor for clinical conditions such as depression and somatization, but also a consequence of these disorders (5, 7).

Because of the intertwining of poor sleep quality, depression, and somatization (i.e., the mixed clinical conditions), it is difficult to identify all modifiable and non-modifiable risk factors. The recent reports categorize the risk factors of mixed clinical conditions as biological, physiological, environmental, family/social, and psychological factors (2, 8). For example, several reports have addressed the importance of biological factors such as sex differences in the pure and mixed clinical conditions (9, 10). Several other reports have attempted to identify the psychological mechanisms associated with pure clinical conditions (5, 11, 12). Of course, the aim of these studies was mainly to examine non-personality than personality pathology (7, 12, 13).

More traditional frameworks of personality pathology, such as the fourth edition of the Diagnostic and Statistical Manual of Mental Disorders (DSM-IV; 14), were conceptualized under a categorical approach to the measurement of personality disorder (PD). The later version of the manual (i.e., the DSM-5), although it proposed an Alternative Model for Personality Disorders (AMPD) in Section III, retained the previous ten categories of PD including paranoid, schizoid, schizotypal, antisocial, borderline, narcissistic, histrionic, avoidant, dependent, and obsessive-compulsive PDs (14, 15). The associations between these PDs, which are grouped into three clusters A, B, and C, and other psychiatric disorders were previously extensively investigated (16, 17). For example, review studies addressed the associations between Cluster C PDs and pure clinical conditions (16, 17). However, few studies have attempted to address the associations between personality pathology according to the AMPD and pure clinical conditions (18–20). The AMPD includes two Criteria A and B, the first one covers personality functioning and the second contains maladaptive personality traits and domains (15). Personality functioning consists of both intrapersonal (identity and self-direction) and interpersonal (intimacy and empathy) dysfunctions (21). The maladaptive domains of Criterion B include negative affectivity, detachment, antagonism, disinhibition, and psychoticism, which consist of 25 specific traits including anxiousness, emotional liability, separation insecurity, depressivity, submissiveness, rigid perfectionism, perseveration, withdrawal, anhedonia, intimacy avoidance, restricted affectivity, suspiciousness, manipulativeness, grandiosity, deceitfulness, callousness, hostility, attention-seeking, distractibility, irresponsibility, impulsivity, risk-taking, perceptual dysregulation, unusual beliefs/experiences, and eccentricity (22). By discarding the traditional four categories of paranoid, schizoid, histrionic, and dependent, the AMPD also introduces six PD composites (14). Recent review studies provide initial empirical support for the validity of AMPD across cultures (23–27).

Although a recent review (28) found that there is poor knowledge of the associations between the constructs of AMPD and somatization, research has shown that negative affectivity and psychoticism are positive predictors of somatization while antagonism and detachment are negative predictors (29, 30). Negative affectivity, detachment, and disinhibition are positive predictors of depression while antagonism is a negative predictor (30, 31). Negative affectivity positively and antagonism negatively also predict poor sleep quality (30). Although this evidence supports the relationship between maladaptive personality traits and three clinical conditions, personality has a dynamic nature in interaction with environmental and biological (e.g., gender) factors (32). For instance, negative affectivity, which covers anxiousness and emotional liability, leads to somatization and depression, which in turn leads to sleep problems (33, 34). This situation can be more complicated in interaction with gender, since women and men are affected differently by all these clinical conditions (35). We wanted to know how maladaptive personality traits according to the AMPD in interaction with gender predict three dependent variables including poor sleep quality, depression, and somatization.

### The current research

Although there is little knowledge about the association between personality pathology from the perspective of AMPD and some clinical conditions such as sleep disorders (20, 36), previous efforts to identify links between comprehensive psychological factors such as PDs and the mixed clinical conditions had some serious limitations for several reasons (20, 28, 29, 31). First, although associations between PDs or maladaptive personality factors and pure clinical conditions (i.e., poor sleep quality or depression or somatization) were reported by some studies (20, 29, 31, 36), these psychiatric conditions are usually mixed or comorbid problems (3–6). Second, the mediating role of biological factors such as sex differences in the relationship between maladaptive personality factors and mixed clinical conditions was neglected while all the clinical conditions are affected by biological gender (9, 10). Third, previous studies have attempted to report the relationship between non-PDs such as anxiety disorders and pure clinical conditions (13). Although non-personality pathology is more closely related to poor sleep quality and somatization (5), PDs are associated with many psychiatric disorders (37, 38). Fourth, previous studies aimed to report the associations between PDs or normal personality traits and pure clinical conditions (28, 39). Therefore, few research reported the associations between maladaptive personality traits proposed in Section III of DSM-5 and the mixed clinical conditions. Fifth, the dynamic nature of personality traits, despite relative stability, is affected by environmental situations (32, 40). Previous reports mainly focused on the cross-sectional and longitudinal associations between personality and psychiatric disorders without considering the moment-to-moment dynamics of personality in response to environmental situations (5, 17–19, 39). And finally, previous reports did not consider a deep machine learning network to cover all dynamic maladaptive personality traits and sex differences associated with mixed clinical conditions (41, 42).

According to these considerations, we wanted to know how maladaptive personality traits (i.e., independent variables) are linked to mixed clinical conditions (i.e., dependent variables) when sex differences are involved. However, it is very difficult to manage and analyze large and mixed health data using traditional approaches. Models for Artificial Neural Network (ANN) analysis can be useful in such cases for several reasons (e.g., the ability to manage high-dimensional problems and reduce the possibly complex nature of results and provide concrete advice for practitioners) listed in a previous report (43). Recent reports also address the opportunities for applying machine learning to personality measurement (44–47). Therefore, the present study was conducted with two objectives. Our first purpose was to classify the participants based on the dimensional measures of all three clinical conditions (somatization, depression, and poor sleep quality) using the cluster analysis method. The cluster analysis helps to form groups with mixed conditions based on maximum similarity within the groups and maximum difference between the groups. Second, we aimed to predict the derived clusters by maladaptive personality traits and sex differences using a deep machine learning approach.

## Method

### Design and samples

A sample of 900 young adults aged 18-40 years from Kermanshah, western Iran, consented to participate in this cross-sectional study. The samples were invited to participate in a survey by mobile phone applications from September to December 2022. In case of initial agreement and providing consent to participate in the study, we checked the inclusion criteria. Thus, all participants were asked to be free from substance abuse and any psychiatric medication in the last four weeks. As these were the inclusion criteria, we asked these individuals not to participate in the study. Additionally, we requested that only individuals aged 18–40 complete the questionnaires. Then, we sent a link to the electronic questionnaires privately to each of the eligible participants. We used mobile invitations and online links to collect data for several reasons, including not limiting the sample to people available in the community (e.g., students and working people), saving participants’ time, avoiding unnecessary transports and trips by data collectors and participants, directly entering the answers into the statistical software and saving the time of the research team, and saving the costs of transportation and pencil-paper questionnaires. Additionally, this method facilitated the participation of those who might not cooperate due to fear of the coronavirus and social distancing. Exclusion criteria were incomplete questionnaires or missing data for more than 20% of the items, which were checked when data collection was completed.

There were 848 (94%) questionnaires returned to the research team, 9 of which were excluded due to many missing items. The final sample contained 839 people aged 18 to 40 years (28.7 ± 6.3). The participants were mostly female (n = 538, 64%), single (n = 473, 56%), university-educated (n = 527, 63%), and college students (n = 260, 31%). The data was collected using the Persian versions of the PID-5 (220 items; 22, 48), the Revised Form of Symptom Checklist-90 (SCL-90-R; 49), and sleep quality subscale of Scale for Pseudo-Cardiac Symptoms and Poor Sleep Quality (SPSQ: 4 items; 5). The PID-5 was used to measure independent variables while the SCL-90-R and SPSQ were used to measure dependent variables. This study was approved by the ethics committee of an academic institute (ID: IR.KUMS.REC.1402.125) and follows the declaration of Helsinki.

### Data measurement

#### Personality inventory for DSM-5

The PID-5 is a 220-item self-report questionnaire used to assess 25 facets of personality and five maladaptive domains according to AMPD Criterion B (22, 48). The facets are included depressivity, callousness, risk-taking (all 14 items), eccentricity (13 items), perceptual dysregulation (12 items), withdrawal, deceitfulness, hostility, rigid perfectionism (all 10 items), anxiousness, distractibility, perseveration (all 9 items), anhedonia, unusual beliefs & experiences, attention-seeking (all 8 items), emotional liability, separation insecurity, irresponsibility, restricted affectivity, suspiciousness (all 7 items), intimacy avoidance, grandiosity, impulsivity (all 6 items), manipulativeness (5 items), and submissiveness (4 items). The maladaptive domains consist of negative affectivity, disinhibition, antagonism, detachment, and psychoticism. Items response is based on a Likert scale for often false to often true (ranging from 0 to 3). The reliability and validity of the PID-5 among clinical and nonclinical Persian language samples are acceptable *(* 50, 51). Cronbach’s alpha for the total scale was acceptable in the present study (*α* = .97).

#### Revised form of symptom checklist-90

This 90-item checklist was developed to assess the symptoms of mental disorders (49). Nine clinical subscales include depression (13 items), somatization (12 items), anxiety, obsessive-compulsive disorder, psychoticism (all 10 items), interpersonal sensitivity (9 items), phobic anxiety (7 items), hostility, and paranoid ideation (both 6 items). This SCL-90-R also includes seven additional items. The answers to each item are graded based on a five-point Likert scale from zero (no discomfort) to four points (very severe discomfort). The reliability and validity of the SCL-90-R among Persian language samples are acceptable (52). Only depression and somatization subscales are used in this study, and their Cronbach’s alpha in the present sample were .91 and .89, respectively.

#### Scale for pseudo-cardiac symptoms and poor sleep quality

This 11-item scale was designed to evaluate both pseudo-cardiac symptoms (7 items) and poor sleep quality (4 items). All items were adapted from three standard questionnaires related to the content of the scale. Items 1-5, 7, and 8 were used to measure pseudo-cardiac symptoms while items 6 and 9-11 were used to measure sleep quality. The items are graded based on a five-point Likert scale from zero (no discomfort) to four points (very severe discomfort). The reliability and validity of the SPSQ among Persian language samples are acceptable (5). Only the sleep quality subscale is used in the present study. Cronbach’s alpha for the sleep quality subscale was relatively acceptable in our sample (*α* = .77).

### Analytic plan

The hierarchical and *K*-mean cluster analysis techniques were used to cluster the scores of dependent variables including sleep quality, depression, and somatization. First, a hierarchical clustering method with Squared Euclidean Distance was used to identify the number of clusters. The centroid clustering method for models including 2 to 6 clusters was independently evaluated with the highest discriminative power for the four-cluster model. Then, we used the *K*-means clustering method to determine the four clusters suggested by the initial analysis. The stability of the cluster solution structure and concordance among solutions were checked using Cramer’s *V*. We reported the mean and standard deviation of both the clustering factors and all maladaptive traits. The maladaptive traits scores and biological gender distribution, which were independent variables in the present study, between clusters were compared by the Analysis of Variance (ANOVA) and Chi-square tests, respectively. We checked the assumptions (e.g., normality and homogeneity of variance) of parametric statistics before applying ANOVA. For head-to-head comparisons of the clusters, we used Tukey’s *post hoc* test.

In the next step, we used the ANN modeling to investigate the complex connections between all independent variables (the maladaptive traits and biological gender) and the dependent variables (i.e., the identified clusters). The ANN was used to test the predictability of the clusters by both maladaptive traits and gender. The subjects’ gender was entered into the model because it is a highly influential factor in all clinical conditions included in the cluster analysis (9, 10). However, ANN is also a useful approach when the model includes nonlinear multiple variables (53). We used a multilayer perceptron (MLP) that is a fully connected class of feedforward ANN. The MLP which consists three types of input, output, and hidden layers learns the relationships between linear and non-linear data. Then, a receiver operating characteristic (ROC) curve of independent variables with high sensitivity specific to the identified clusters was performed. We reported an Area under the ROC Curve (AUC) for ROC Curve of all four identified clusters, where values equal to .80 and above indicate a model with the excellent predictive ability (54). All analyses were performed using the SPSS software and *p* ≤.05 was considered the significance level.

## Results

### Clustering of dependent variables

The hierarchical and *K*-mean cluster analysis techniques suggested a four-cluster solution. The agreement coefficient calculated indicated that the cluster solution structure in both models is fully stable, and an acceptable concordance is observed across solutions (Cramer’s *V = .*522, *p* <.001). **Table 1** shows the identified clusters according to the included variables. As can be seen, all clusters are significantly different in all three clustering factors including poor sleep quality, depression, and somatization (*p* <.001). According to the score of the variables included in the *K*-mean cluster analysis, the labels of each cluster were selected (I: Depressed Poor Sleepers, II: Nonclinical Good Sleepers, III: Subclinical Poor Sleepers, IV: Clinical Poor Sleepers).

**Table 1:** The identified clusters according to the included variables and the results of ANOVA for differences in all maladaptive traits across the clusters.

Variables	Total sample (*N* = 839)	Cluster I: Depressed poor sleepers (*N* = 138, 16.4%)	Cluster II: Nonclinical good sleepers (*N* = 406, 48.4%)	Cluster III: Subclinical poor sleepers (*N* = 178, 21.2%)	Cluster IV: Clinical poor sleepers (*N* = 117, 14.0%)	F	*P*	Tukey *post hoc* test
Clustering factors								
Poor sleep quality	4.43 ± 3.63	5.29 ± 3.45	2.27 ± 2.47	6.37 ± 3.09	7.96 ± 3.25	165.68	<.001	II < I < III < IV
Somatization	13.66 ± 9.66	11.73 ± 4.30	6.35 ± 4.22	22.14 ± 4.37	28.38 ± 6.35	944.15	<.001	II < I < III < IV
Depression	17.16 ± 11.27	25.54 ± 6.19	7.73 ± 4.63	20.50 ± 4.66	34.91 ± 5.96	1074.97	<.001	II < III < I < IV
Comparative factors								
Maladaptive traits								
Emotional liability	1.27 ±.56	1.47 ±.57	1.08 ±.53	1.37 ±.47	1.57 ±.55	39.36	<.001	II < I, III, IV; III < IV
Anxiousness	1.21 ±.61	1.54 ±.62	.95 ±.52	1.28 ±.49	1.66 ±.60	76.95	<.001	II < III < I, IV
Separation insecurity	1.02 ±.59	1.15 ±.62	.86 ±.57	1.08 ±.51	1.34 ±.57	26.74	<.001	II < I, III < IV
Withdrawal	1.02 ±.54	1.15 ±.58	.86 ±.52	1.08 ±.50	1.32 ±.48	29.37	<.001	II < I, III < IV
Anhedonia	1.11 ±.55	1.40 ±.53	.86 ±.46	1.17 ±.45	1.55 ±.51	87.36	<.001	II < III < I, IV
Intimacy avoidance	1.01 ±.59	.99 ±.61	.91 ±.55	1.15 ±.59	1.21 ±.60	12.21	<.001	II < III, IV
Manipulativeness	.95 ±.52	.93 ±.56	.89 ±.52	1.08 ±.48	.98 ±.52	5.63	.001	II < III
Deceitfulness	.89 ±.54	.92 ±.56	.76 ±.53	1.00 ±.51	1.12 ±.47	19.18	<.001	II < I, III, IV; I < IV
Grandiosity	1.18 ±.54	1.17 ±.57	1.14 ±.55	1.27 ±.51	1.22 ±.54	2.30	.076	I = II = III = IV
Irresponsibility	.86 ± 54	.86 ±.50	.73 ±.51	1.00 ±.55	1.14 ±.51	24.30	<.001	I, II < III, IV
Impulsivity	.98 ±.65	1.05 ±.68	.79 ±.62	1.07 ±.56	1.43 ±.56	36.30	<.001	II < I, III < IV
Distractibility	1.07 ±.54	1.25 ±.58	.88 ±.49	1.12 ±.49	1.46 ±.47	49.63	<.001	II < I, III < IV
Unusual Beliefs	.89 ±.55	.84 ±.55	.75 ±.51	1.11 ±.52	1.07 ±.56	25.57	<.001	I, II < III, IV
Eccentricity	.91 ±.61	.98 ±.64	.72 ±.58	1.06 ±.55	1.25 ±.55	32.02	<.001	II < I, III < IV
Perceptual dysregulation	.82 ±.48	.86 ±.46	.62 ±.45	.98 ±.39	1.21 ±.39	67.46	<.001	II < I, III < IV
Attention seeking	1.27 ±.57	1.35 ±.61	1.20 ±.59	1.27 ±.48	1.40 ±.59	4.84	.002	II < I, IV
Callousness	.80 ±.51	.78 ±.51	.68 ±.48	.97 ±.51	.99 ±.48	5.18	<.001	I, II < III, IV
Depressivity	.89 ±.61	1.16 ±.55	.58 ±.50	.96 ±.50	1.52 ±.51	122.91	<.001	II < III < I < IV
Hostility	1.18 ±.52	1.33 ±.55	1.03 ±.51	1.24 ±.45	1.43 ±.45	26.46	<.001	II < I, III, IV; III < IV
Perseveration	1.13 ±.51	1.27 ±.49	.96 ±.49	1.21 ±.46	1.43 ±.47	36.77	<.001	II < I, III < IV
Restricted affectivity	1.07 ±.53	1.16 ±.53	.95 ±.50	1.15 ±.52	1.23 ±.56	14.14	<.001	II < I, III, IV
Rigid perfectionism	1.33 ±.49	1.46 ±.47	1.25 ±.49	1.35 ±.44	1.44 ±.51	9.51	<.001	II < I, IV
Risk-taking	1.38 ±.42	1.24 ±.42	1.37 ±.43	1.45 ±.38	1.46 ±.36	9.01	<.001	I < II, III, IV
Submissiveness	1.15 ±.57	1.30 ±.57	.99 ±.55	1.21 ±.53	1.39 ±.53	22.89	<.001	II < I, III, IV; III < IV
Suspiciousness	1.35 ±.45	1.53 ±.47	1.20 ±.42	1.40 ±.41	1.57 ±.44	35.92	<.001	II < I, III, IV; III < I
Gender, female	538 (64.1%)	100 (11.9%)	234 (27.9%)	122 (14.5%)	82 (9.8%)	14.92*	.002	Na

*indicates the results of Chi-square test.

ANOVA, Analysis of variance; Na, not applicable.

### Comparison of the independent variables between the clusters

**Table 1** also shows the results of ANOVA for the differences in all maladaptive traits across the clusters. The results of this table show that the scores of all maladaptive personality traits (except grandiosity) are significantly different between the identified clusters (All *F* between 4.84 and 122.91, all *p* ≤.002). Compared to Cluster II (Nonclinical Good Sleepers), other clusters show a severe clinical profile on all maladaptive traits except grandiosity (all *p* <.05). Exceptionally, the score of risk-taking in Cluster I (Depressed Poor Sleepers) was significantly higher than the score of Cluster II (Nonclinical Good Sleepers). Also, the number of women and men is significantly different among the clusters (*χ^2^* = 14.918*, p* = .002).

### Prediction of the clusters by the independent variables

Using the ANN model, we tested the predictability of the identified clusters using all maladaptive traits and subjects’ gender. **Table 2** shows the summary of the ANN model and statistics of the hidden layer and units. A number of 594 (70.8%) and 245 (29.2%) of all dataset samples were selected to create the training and testing samples, respectively. The model containing 27 independent variables and four dependent variables led to the identification of one hidden layer with two hidden units. The overall bias for each of the first to fourth clusters was equal to .52, .63, .20, and −.97, respectively.

**Table 2:** Summary of the ANN model and statistics of the hidden layer and unites.

The model summary	The identified clusters
Training	
Number of subjects (%)	594 (70.8)
Cross entropy error	553.10
Incorrect predictions (%)	39.1
Testing	
Number of subjects (%)	245 (29.2)
Cross entropy error	243.55
Incorrect predictions	41.6
Number of units	
Independent variable	27
Dependent variable	4
Hidden layer	2
Input layer	
Unit 1 bias	−.95
Unit 2 bias	.44
Output layer	
Depressed poor sleepers (bias)	.52
Hidden unit 1	.88
Hidden unit 2	−.34
Nonclinical good sleepers (bias)	.63
Hidden unit 1	−1.35
Hidden unit 2	−1.63
Subclinical poor sleepers (bias)	.20
Hidden unit 1	−.51
Hidden unit 2	−.21
Clinical poor sleepers (bias)	−.97
Hidden unit 1	1.04
Hidden unit 2	1.79

Number of layers for all dependent variables = 1, Activation function of hidden layer = Hyperbolic Tangent, Error function = Cross-entropy, Rescaling method for covariates = Standardized.

### Sensitivity analysis of the independent variables to detect the clusters

**Figure 1:**
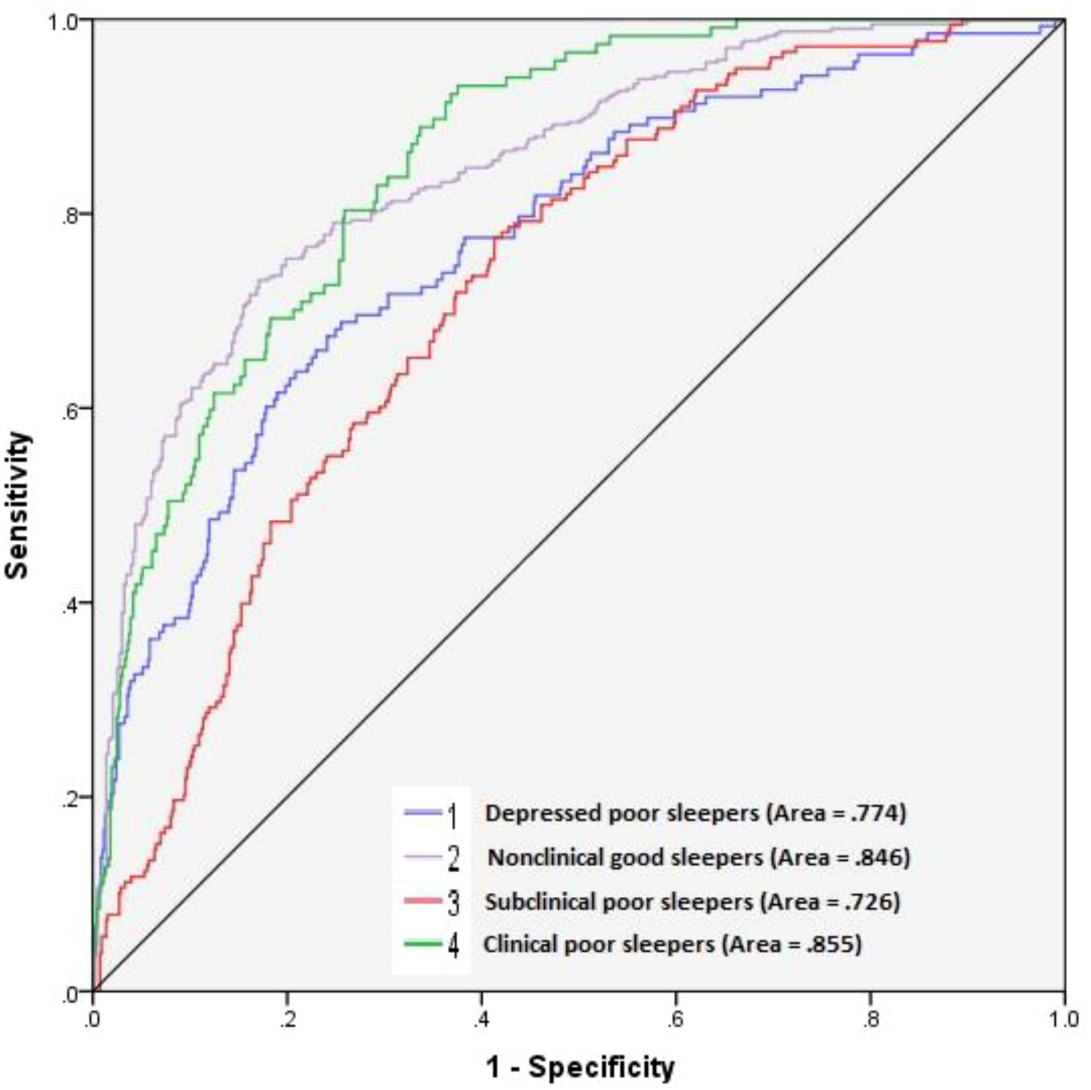
ROC curve of maladaptive traits and gender with high sensitivity specific to the identified clusters.

**Figure 1** shows the ROC curve of maladaptive traits and gender with high sensitivity specific to the identified clusters. As can be seen, all the ROC curves across the clusters of the dependent variable are far away from the diagonal reference line. The AUC for ROC Curve for both Clinical Poor Sleepers (= .855) and Nonclinical Good Sleepers (= .846) is completely acceptable. This means that the neural network can predict clinical or non-clinical subjects with a sensitivity of 85 percent. The AUC for ROC Curve both Depressed Poor Sleepers (= .774) and Subclinical Poor Sleepers (= .726) is also relatively acceptable. Overall, the ROC curve supports the results obtained by the ANN model.

### Importance analysis of the independent variables to detect the clusters

**Table 3** shows the importance and normalized importance of all independent variables including the maladaptive traits and subjects’ gender. The results of this table show that anhedonia (.92), perceptual dysregulation (.092), depressivity (.083), anxiousness (.071), and unusual beliefs (.069) are very strong traits connected to the clusters, where the normalized importance ranges from 74 to 100%. The gender variable shows a relatively weak significance in the model (importance = .022, normalized importance = 24%). The importance of other maladaptive traits included in the model can be seen in **Table 3**.

**Table 3:** Importance and normalized importance for all maladaptive traits and gender.

Independent variables	Importance	Normalized importance (%)
Maladaptive traits		
Anhedonia	.092	100
Perceptual dysregulation	.092	99.4
Depressivity	.083	89.7
Anxiousness	.071	76.9
Unusual Beliefs	.069	74.2
Withdrawal	.056	60.3
Attention seeking	.051	55.6
Risk-taking	.043	46
Eccentricity	.042	45.3
Intimacy avoidance	.041	44.6
Perseveration	.039	42.3
Emotional liability	.031	33.6
Grandiosity	.031	33.3
Restricted affectivity	.028	30.2
Impulsivity	.028	30.2
Deceitfulness	.026	28.7
Submissiveness	.023	24.7
Hostility	.022	23.8
Suspiciousness	.022	23.8
Irresponsibility	.021	23.1
Callousness	.019	20.3
Rigid perfectionism	.016	17
Distractibility	.015	15.8
Manipulativeness	.012	12.6
Separation insecurity	.007	7.2
Gender	.022	24

## Discussion

The present study aimed to both cluster the samples according to three mixed clinical conditions (i.e., poor sleep quality, depression, and somatization) and to predict the derived clusters by maladaptive personality traits and sex differences using a deep machine learning approach. The cluster analysis techniques suggested a fully stable and acceptable four-cluster solution for Depressed Poor Sleepers, Nonclinical Good Sleepers, Subclinical Poor Sleepers, and Clinical Poor Sleepers. These results address the complexity and intermingling of all three clinical conditions including poor sleep quality, depression, and somatization in the present sample. Some reports show that these clinical conditions are usually mixed or comorbid problems (3–6). At the same time, these clinical conditions are both a risk factor and a health consequence of each other (5, 7).

We found significant differences in all maladaptive personality traits across the clusters. Compared to Cluster II (healthy samples), other clusters show a severe clinical profile on all maladaptive traits except Grandiosity. The most severe clinical profile of maladaptive personality traits was found in Cluster IV (clinical samples). This finding highlights the correlation and overlap between personality pathology and other psychiatric disorders. Although little is known about the links between personality pathology and mixed clinical conditions, several studies have reported associations between PDs and pure clinical conditions (16, 17). Other studies have attempted to report associations between personality pathology according to the AMPD and pure clinical conditions (18–20, 28, 29, 31). A study attempted to test the links between maladaptive personality from the perspective of AMPD and the mixed clinical conditions obtained from factor analysis techniques, the results of which showed a significant relationship (39).

We aimed to predict four clusters derived from maladaptive personality traits using deep machine learning. We also included the gender differences of the samples in the nonlinear prediction model. The ANN model led to the identification of one hidden layer with two hidden units with AUC relatively to completely acceptable for all healthy and unhealthy clusters. Therefore, the present neural network could predict both Clinical Poor Sleepers and Nonclinical Good Sleepers with a sensitivity of 85 percent. Because predictive models with values equal to 80 percent and above are completely acceptable (54), we conclude that machine learning is a practical approach to predict both clinical and non-clinical samples by maladaptive personality traits. The present findings also showed that the predictive model is relatively acceptable for both the Depressed Poor Sleepers and Subclinical Poor Sleepers with a sensitivity equal to or higher than 73 percent. However, applying a machine learning approach to personality assessment can provide an opportunity to access the complex dynamic structures involved in general psychology (32, 46, 47). Recent reports have applied machine learning approaches to personality measurements connected to mental health constructs (41, 42, 55). However, the research body mainly tries to predict the adaptive and maladaptive personality through other structures such as networks and social applications (41, 42, 55). Regardless of these issues, the use of machine learning approaches to measurements of normal and abnormal personality has been recommended since the 1990s (44, 45).

We found that anhedonia, perceptual dysregulation, depressivity, anxiousness, and unusual beliefs are the most valuable traits with importance higher than 70%. All these maladaptive traits are the lower-order factors of the detachment, psychoticism, and negative affectivity domains in the AMPD (14, 22). We conclude from these findings that all domains of AMPD except antagonistic and disinhibited externalizing play a remarkable role in mixed clinical conditions. A previous report supports strong associations between the detachment, psychoticism, and negative affectivity domains of AMPD and the mixed clinical conditions (39). Additionally, some studies addressed the associations between these maladaptive personality domains and three clinical conditions including somatization, depression, and poor sleep quality independently (29–31). The current approach to the hierarchical classification of psychopathology also addresses the mixed clinical conditions associated with these three domains than antagonistic and disinhibited externalizing domains at the spectrum level (56). However, the high value of the maladaptive traits of the AMPD to predict the mixed clinical conditions was effective on the milder importance of gender differences in the present study. This means that abnormal personality, compared to biological gender, is more involved in the psychopathology of mixed clinical conditions. However, we should not neglect the interaction between biological gender and personality dynamics affecting general psychopathology.

### Strengths and Limitations

Our report is a pioneering study to predict the mixed clinical conditions by maladaptive personality traits from the AMPD perspective using ANN modeling. Despite not using a categorical measure to identify cases with poor sleep quality, depression, and somatization, we differentiated a range of healthy to unhealthy groups using cluster analysis techniques. Cluster analysis is an important analytical method for improving clinical practice by identifying subgroups within a larger sample (57). When used appropriately, cluster analysis is a practical approach to mental health reporting (58). However, identifying general populations with multiple psychiatric disorders at the same time using conventional diagnostic interviews is very time-consuming and expensive. Because current psychopathology tends toward transdiagnostic constructs (56), transdiagnostic constructs of personality linked to mixed clinical conditions may help provide clearer boundaries. The young population included in the present study is another strength when some reports suggested that somatization and sleep problems are more common among young than elderly groups (59, 60).

The present study also faced some limitations. We did not include in the study clinical samples with established clinical disorders. Although we used dimensional measurement and the current dimensional approach to measuring psychological constructs is not only specific to categorized patients (61), the reproducibility of the analyses and results in clinical inpatients and outpatients is necessary for the generalizability of the present results. We used self-reported scores to measure symptoms of all clinical conditions and maladaptive personality traits. Although this is a common subjective measurement approach in psychological studies (62), future studies can test the validity and stability of the collected data in agreement with other sources of information (at least for maladaptive personality traits). We measured poor sleep quality using a very short standardized scale. The measurement using the Pittsburgh Sleep Quality Index (PSQI; 63) can test the validity of part of the processes and results. We think that including both normal and abnormal range personality traits in ANN in future studies can provide models with more robust learning.

## Conclusion

The cluster analysis techniques address the complexity of three clinical conditions including poor sleep quality, depression, and somatization by identifying one non-clinical cluster and three clinical clusters. The ANN model led to the identification of one hidden layer with two hidden units with AUC relatively to completely acceptable for all healthy and unhealthy clusters. The present findings showed that a machine learning approach can be well used to predict mixed clinical conditions by maladaptive personality traits. In conclusion, this study significantly contributes to our understanding of the interplay between personality traits and clinical conditions. Future research should incorporate diverse populations, longitudinal designs, and clinical samples to validate and extend these findings. Future research also can test the complexity of normal personality traits connected to mixed clinical conditions using machine learning methods.

## Data availability statement

The raw data supporting the conclusions of this article will be made available by the authors, without undue reservation.

## Ethics statement

The studies involving humans were approved by Kermanshah University of Medical Sciences. The studies were conducted in accordance with the local legislation and institutional requirements. The participants provided their written informed consent to participate in this study.

## Author contributions

HK: Conceptualization, Funding acquisition, Investigation, Project administration, Supervision, Validation, Visualization, Writing – original draft. FR: Conceptualization, Investigation, Supervision, Validation, Visualization, Writing – review & editing. AZ: Conceptualization, Data curation, Methodology, Project administration, Resources, Validation, Visualization, Writing – review & editing. BF: Conceptualization, Data curation, Investigation, Project administration, Resources, Visualization, Writing – review & editing. SK: Conceptualization, Formal Analysis, Funding acquisition, Investigation, Methodology, Project administration, Resources, Software, Validation, Visualization, Writing – original draft.
